# Associations between weight stigma and mental well-being among people in romantic relationships: an actor-partner interdependence model investigation

**DOI:** 10.3389/fpsyt.2025.1576406

**Published:** 2025-06-06

**Authors:** Paula M. Brochu, Emily J. Georgia, Madeline Jubran, Molly Robbins, Katherine West, Jillian Crocker, Alexandria M. Schmidt, Katerina Rinaldi, Em Joseph, McKenzie K. Roddy

**Affiliations:** ^1^ Department of Clinical and School Psychology, Nova Southeastern University, Fort Lauderdale, FL, United States; ^2^ Department of Medicine, Vanderbilt University Medical Center, Nashville, TN, United States

**Keywords:** weight stigma, mental well-being, actor-partner interdependence model, perceived weight discrimination, internalized weight bias, weight stigma concerns, romantic relationships

## Abstract

**Background:**

Romantic relationships are primary sources of mental well-being, including life satisfaction. Stigma not only has adverse effects on individual mental well-being but also negatively affects relationship functioning. The purpose of this dyadic, cross-sectional study was to examine the associations between internalized, anticipated, and experienced weight stigma and mental well-being among people in romantic relationships and their partners.

**Method:**

Prolific, an online crowdsourcing platform, was used to recruit 287 couples in long-term relationships who resided in the United States. Participants completed measures of internalized weight stigma, anticipated weight stigma, experienced weight stigma, and mental well-being. Actor-partner interdependence models estimated the associations between participants’ weight stigma and their own mental well-being (actor effect) and the mental well-being of their romantic partners (partner effect).

**Results:**

As expected, significant negative associations were observed between participants’ internalized, anticipated, and experienced weight stigma and their own mental well-being; these actor effects had small to medium effect sizes. Significant negative associations were also observed between participants’ internalized and anticipated weight stigma and their partners’ mental well-being; these partner effects had small effect sizes. Unexpectedly, a significant partner effect was not observed for experienced weight stigma.

**Conclusions:**

Weight stigma is negatively associated with individual mental well-being as well as the mental well-being of romantic partners. Future research is needed to replicate and expand these findings and examine internalized and anticipated weight stigma as potential mechanisms through which experienced weight stigma may affect mental well-being among people in romantic relationships and their partners.

## 1 Introduction

The quality of romantic relationships is one of the strongest predictors of mental health and well-being: People in highly satisfied relationships also report higher life satisfaction (1). Stigma and discrimination negatively affect romantic relationship quality for members of stigmatized groups, including those experiencing barriers based on race, ethnicity, and sexual identity (2–7). Weight stigma, particularly weight criticism from romantic partners, is negatively associated with relationship functioning (8, 9). Thus, not only is stigma in general, including weight stigma in particular, negatively associated with mental health broadly (10–12), it has the potential to also be negatively associated with the mental health of romantic partners. Although stigma is considered a relational stressor, the impact of weight stigma on the mental health of romantic relationship partners is less well understood. The purpose of the current study is to examine the dyadic associations between internalized, anticipated, and experienced weight stigma and mental well-being among people in romantic relationships and their partners.

### 1.1 Romantic relationships and mental health

Romantic relationships play a vital role in the human experience, as they influence people’s sense of identity and well-being (13, 14). Relationship satisfaction is one of the greatest predictors of quality of life, such that people in more fulfilling relationships are more satisfied with their life overall (1). Evidence suggests a link between romantic relationships and mental health, such that partners in satisfying relationships experience improved mental well-being (15). Relationship satisfaction is associated with better emotional and mental health, as higher satisfaction is correlated with happiness, reduced emotional distress, and lower rates of psychotic symptoms (16–18).

Conversely, relationship distress deteriorates functioning and well-being on individual, familial, and societal levels. Couples in unsatisfying romantic relationships display more anger, criticism, and disgust than those in satisfied partnerships (19). A literature review analyzing nationally representative samples of married adults in the United States reveals that unsatisfied relationships are correlated with an increased probability of suicidality and suicide attempts, as well as anxiety, eating, substance use, and personality disorders (20). Relationship distress is a prominent presenting problem in individual therapy and its presence buffers the impact of treatment for other psychological concerns, such as depressive and anxiety disorders (21). Furthermore, several physical health ailments are also associated with unsatisfying romantic relationships, including greater risk for coronary heart disease, lower immunity, and premature mortality (22).

### 1.2 Stigma, relationship quality, and mental health

Stigma has adverse effects on both mental health and romantic relationship functioning (2, 3, 6, 11, 23). Stigmatization refers to social devaluation of a person or group due to the perception of characteristics as socially disadvantageous in a particular power structure (24). Encounters with discrimination represent just one component of stigma (25, 26). Internalized, anticipated, and experienced stigma constitute a multifaceted conceptualization of the experience of stigma and feeling stigmatized. Whereas experienced stigma refers to the discrimination a person has experienced or perceived, anticipated stigma involves concern over being treated unfairly. Internalized stigma involves the application of negative stereotypes to the self and self-derogation.

A meta-analysis of 49 empirical studies found a significant positive association between experiencing discrimination based on a variety of characteristics (e.g., race/ethnicity, gender, sexual identity, mental and physical illness) and mental health conditions including depression, anxiety, psychosis, psychological distress, and loneliness as well as lower self-esteem, quality of life, happiness, life satisfaction, and well-being (11). However, variation in the strength of mental health associations depending on the type of stigma was emphasized, such that associations were stronger for physical illness-related stigmas than mental illness-related stigmas, with social stigmas falling in the middle.

Regarding relationship quality, a meta-analysis of 35 empirical studies shows that experiencing discrimination on the basis of sexual identity is negatively associated with relationship quality, including indicators of passion, relationship satisfaction, intimacy, support, commitment, and trust (3). Additional studies document the negative association between experienced stigma on the basis of race/ethnicity, gender, and age and relationship quality (2, 4–6, 23). Of particular interest, emerging research demonstrates that experienced stigma not only negatively affects individual mental health, but also the mental health of romantic partners (7, 27). Everyday experiences of discrimination, particularly on the basis of gender, race, and age, are negatively associated with depression for people in romantic relationships as well as their partners, an effect mediated by relationship strain (7). In couples consisting of transgender women and cisgender men, experienced stigma is associated with elevated psychological distress for both partners, an effect attenuated by relationship commitment for transgender women but not their cisgender male partners (27).

In a systematic review of 83 studies examining associations between internalized stigma, anticipated stigma, and depression, internalizing and anticipating stigma on the basis of gender, sexual identity, weight, and physical illness were positively associated with depression (28). Internalizing stigma on the basis of sexual identity is negatively associated with relationship functioning and demonstrates a larger effect size than that between perceived discrimination and relationship functioning (3). To date, previous research has not examined whether and how internalized and anticipated stigma are associated with the mental well-being of romantic partners.

### 1.3 Weight stigma and mental health: a relational perspective

Weight stigma refers to the social devaluation of people who are perceived to exceed socially-constructed weight expectations (29). Weight stigma is a pervasive, harmful, and widespread societal issue that negatively affects mental health. As theorized by Earnshaw and Chaudoir (25), experienced, anticipated, and internalized stigma are central, distinct processes through which stigmatization negatively affects psychological, behavioral, and physical health outcomes. This model is relevant to weight stigma. Experienced weight stigma refers to the discrimination a person has experienced or perceived based on their weight (30). Anticipated weight stigma involves concern over being treated unfairly because of one’s weight (30). Internalized weight stigma involves the application of negative weight stereotypes to the self and self-derogation because of weight (31). Notably, internalized and anticipated weight stigma are theorized to develop through experiences of weight stigma (30, 32), although internalized weight stigma shows even stronger negative effects on health and well-being than experienced weight stigma (33).

In a meta-analysis of 105 empirical studies, Emmer et al. (10) found significant associations between experienced and internalized weight stigma and mental health outcomes, including positive associations with depression, anxiety, psychological distress, and disordered eating, and negative associations with self-esteem, well-being, quality of life, and life satisfaction. Gender did not moderate these findings. Internalized weight stigma had stronger associations with mental health than experienced weight stigma. In a systematic review and meta-analysis of 33 empirical studies, Wu and Berry (12) also found that experienced and internalized weight stigma were positively associated with disordered eating, depression, anxiety, and body dissatisfaction and negatively associated with self-esteem. Although anticipated weight stigma was not included in these analyses, research shows that anticipated weight stigma is positively associated with disordered eating, including dietary restraint, eating concerns, body shape and weight concerns, binge eating, and unhealthy weight control behaviors (34, 78).

There is growing recognition of the relational impact of weight stigma, particularly within romantic relationships (8, 9). Much of this research focuses on romantic relationships as a potent source of weight stigmatization, particularly through expressions of weight criticism between partners. Weight criticism is associated with lower relationship satisfaction and sexual intimacy and heightened relational conflict (9).

Limited research has examined the relational dynamics of internalized, anticipated, and experienced weight stigma outside of weight criticism between romantic partners. Internalized weight stigma is associated with lower relationship satisfaction and sexual intimacy among men and women in heterosexual relationships (35, 36). Experienced weight stigma is associated with lower sexual satisfaction in a sample of predominantly heterosexual Black and White men (37). As such, weight stigma not only harms the individual but also strains interpersonal relationships, potentially impacting the well-being of romantic partners. To date, no research has examined the dyadic associations between weight stigma and mental well-being among people in romantic relationships. Given the relational dynamics at play, examining the associations between weight stigma and mental well-being within the context of romantic relationships is crucial.

### 1.4 Present study

This study sought to examine the dyadic associations between internalized, anticipated, and experienced weight stigma and mental well-being among people in romantic relationships and their partners. In addition to internalized weight stigma, of specific interest were general weight stigma experiences and concerns from other people, rather than inquiring specifically about romantic partners as a source of weight stigma. Utilizing the actor-partner interdependence model (APIM; 38), the associations between participants’ own weight stigma and their mental well-being were examined (actor effects), as well as their partners’ mental well-being (partner effects). Following previous research demonstrating the adverse effects of stigma on mental health (10, 11), it was hypothesized that greater internalized, anticipated, and experienced weight stigma reported by participants would be negatively associated with their own mental well-being. Furthermore, following previous research showing that stigma negatively affects romantic relationships among those experiencing injustice based on race, gender, age, and sexual identity (2, 3, 6, 7), it was hypothesized that greater internalized, anticipated, and experienced weight stigma reported by participants would be negatively associated with the mental well-being of their romantic partners. Potential moderation by participant gender was explored in this study. Women are often thought to be more affected by weight stigma, which has led studies to primarily focus on the consequences of weight stigma on women while leaving men overlooked and understudied (39).

## 2 Method

### 2.1 Participants and procedure

**Figure 1:**
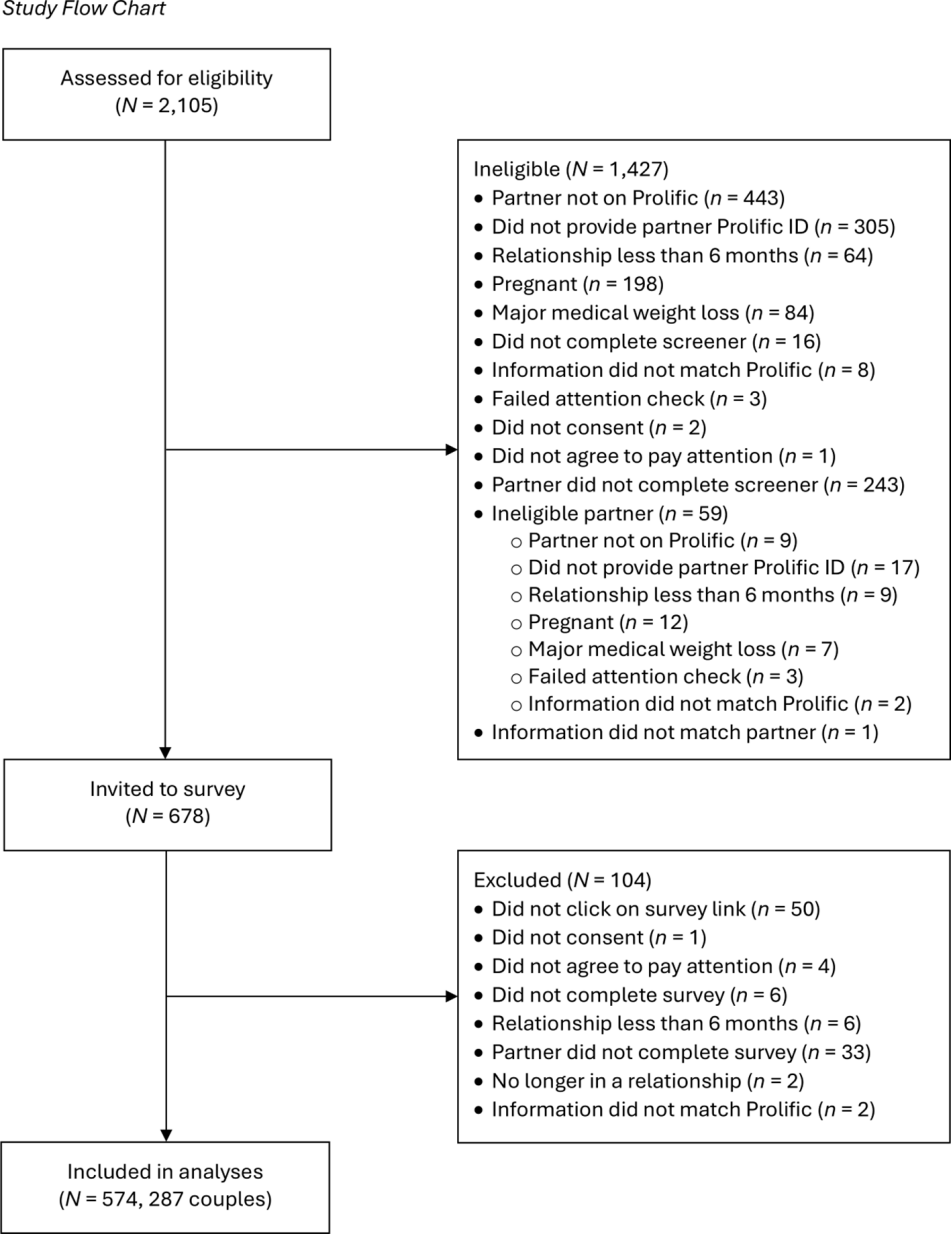
Study Flow Chart.

Participants were recruited through Prolific, an online crowdsourcing platform to collect high-quality data from community members (40). An eligibility screener was used to recruit couples; to participate, Prolific workers had to reside in the United States, have an approval rating of at least 95%, and have at least 100 previous submissions. To be eligible for the study, participants must have had a romantic partner who was also on Prolific, provided a unique and valid Prolific worker ID for their partner, be in a romantic relationship of at least six months, not be pregnant or have given birth in the past year or plan to become pregnant in the next year, and not be experiencing major medical weight loss (e.g., chemotherapy, bariatric surgery) due to potential changes in body size. Participants were compensated US$0.40 for completing the eligibility screener. Eligible couples were then invited to participate in the current study described as examining perceptions of body, weight, and shape in romantic relationships. Participants were compensated US$4.00 for completing the survey. **Figure 1** presents the study flow chart which outlines the details of participant ineligibility and exclusion from the survey data. All decisions regarding participant eligibility and exclusion took place before any data analyses were conducted.

The final sample consisted of 287 couples (259 different-sex, 28 same-sex) comprised of 574 participants (sex: 301 female, 273 male; gender: 289 women, 269 men, 16 gender non-binary) in romantic relationships of at least six months (*M* = 10.77 years, *SD* = 8.45 years). Participants ranged in age from 19 to 76 years (*M* = 39.19, *SD* = 11.80). Most participants identified as White (*n* = 477, 83%); of the remaining participants, 69 (12%) identified as Asian, 57 (10%) as Hispanic or Latine, 28 (5%) as Black or African American, 15 (3%) as American Indian or Alaska Native, 3 (1%) as Native Hawaiian or other Pacific Islander, and 3 (1%) as Middle Eastern. Four participants specified a different racial/ethnic identity; participants could select more than one racial/ethnic identity. Most participants identified as heterosexual (*n* = 436, 76%); of the remaining participants, 58 (10%) identified as bisexual, 33 (6%) as gay/lesbian, 15 (3%) as pansexual, 14 (2%) as queer, 8 (1%) as asexual. Nine participants specified a different sexual identity; one participant did not report their sexual identity. Based on self-reported weight and height, participants’ body mass index (BMI) ranged from 14.52 to 60.68 kg/m^2^ (*M* = 29.44, *SD* = 7.77). On a scale from 1 (*thin, underweight, lower-weight*) to 7 (*fat, overweight, higher-weight*), 86 participants (15%) perceived themselves below the scale midpoint, 140 (24%) at the scale midpoint, and 348 (61%) above the scale midpoint (*M* = 4.76, *SD* = 1.36).

All study procedures were determined exempt from the authors’ Institutional Review Board. This study is part of a larger project examining dyadic, longitudinal associations between weight stigma, relationship functioning, and health. Data and codebook are available on the Open Science Framework (https://osf.io/argzt/?view_only=8bcd35aeeb1145c3aa3454cc580db87e). No studies to date have been published from these data. For the larger project’s primary longitudinal mediation analysis, at least 220 couples were sought for participation. Couples were over-sampled due to attrition concerns. The final sample size of 287 couples is ample to examine a simple APIM, where typically at least 120 dyads are recommended (41). Data were collected between November 2023 and June 2024.

### 2.2 Measures

#### 2.2.1 Internalized weight stigma

To assess internalized weight stigma, participants completed the modified Weight Bias Internalization Scale (31). The scale was modified from Durso and Latner’s (42) Weight Bias Internalization Scale so that it could be completed by people regardless of body size. The scale consists of 11 items (e.g., “I hate myself for my weight”). Participants responded to each item on a Likert scale ranging from 1 (*strongly disagree*) to 7 (*strongly agree*). Higher scores indicate more internalized weight stigma. The scale demonstrated excellent internal consistency in the sample (Cronbach’s alpha = .94).

#### 2.2.2 Anticipated weight stigma

The Weight Stigma Concerns Scale (30) was used to assess anticipated weight stigma. The Weight Stigma Concerns Scale was developed from Pinel’s (43) Stigma Consciousness Questionnaires based on gender, sexual orientation, and race/ethnicity. The scale consists of four items (e.g., “I am afraid that other people will reject me because of my weight”). Participants responded to each item on a Likert scale from 1 (*strongly disagree*) to 7 (*strongly agree*). Higher scores indicate more anticipated weight stigma. The scale demonstrated excellent internal consistency in the sample (Cronbach’s alpha = .97).

#### 2.2.3 Experienced weight stigma

To assess experienced weight stigma, participants completed the Perceived Weight Discrimination Scale (30). The Perceived Weight Discrimination Scale was developed from Williams et al.’s (44) widely used measure of perceived racial discrimination. The scale consists of five items (e.g., “In your lifetime, how often have you been treated differently than others because of your weight?”). Participants responded to each item on a scale from 0 (*never*) to 4 (*all the time*). Higher scores indicate more frequent experiences of weight stigma. The scale demonstrated excellent internal consistency in the sample (Cronbach’s alpha = .96).

#### 2.2.4 Mental well-being

Mental well-being was assessed using the Mental Health Continuum-Short Form (45, 81). The scale was derived from its long-form version that assesses the six dimensions of Ryff’s (46) model of psychological well-being and the five dimensions of Keyes’ (47) model of social well-being (48, 49). The measure consists of 14 items comprising three subscales assessing emotional well-being (three items; e.g., “During the past month, how often did you feel happy”), psychological well-being (six items; e.g., “During the past month, how often did you feel that you liked most parts of your personality”), and social well-being (five items; e.g., “During the past month, how often did you feel that you had something important to contribute to society”). Participants responded to each item on a scale from 0 (*never*) to 5 (*every day*). Higher scores indicate more frequent experiences of mental well-being. The total scale (Cronbach’s alpha = .94) and each subscale (emotional: Cronbach’s alpha = .91; psychological: Cronbach’s alpha = .90; social: Cronbach’s alpha = .88) demonstrated good to excellent internal consistency in the sample.

According to Keyes et al. (45), people can be classified as flourishing or languishing in terms of mental well-being. In order to be flourishing, participants must report that they experience seven of the 14 items from the Mental Health Continuum-Short Form ‘everyday’ or ‘almost every day,’ including one of the emotional well-being items. In order to be languishing, participants must report that they experience seven of the 14 items ‘never’ or ‘once or twice,’ including one of the emotional well-being items. Participants who do not fit these criteria are classified as having moderate mental well-being.

#### 2.2.5 Attention checks

The eligibility screener and the survey informed participants the study required they read the questions carefully and that attention checks would be used to assess whether they are reading the questions attentively. To proceed, participants were required to affirm that they were willing to pay careful attention to the survey. If participants indicated that they were not able to pay careful attention to the survey, they were removed from the survey before completion. One attention check was included in the eligibility screener and three attention checks were included in the survey (e.g., “Please select ‘Agree.’ This item is for verification purposes”).

### 2.3 Data analyses

All preliminary analyses were conducted using SPSS 29.0.1.0 (80). All values of *p* <.05 were considered statistically significant and two-tailed *p* values are reported. None of the scale items had missing values. After reverse-scoring the necessary items, mean scale scores were calculated. Bivariate correlations were used to examine the associations between the variables and determine covariate inclusion.

**Figure 2:**
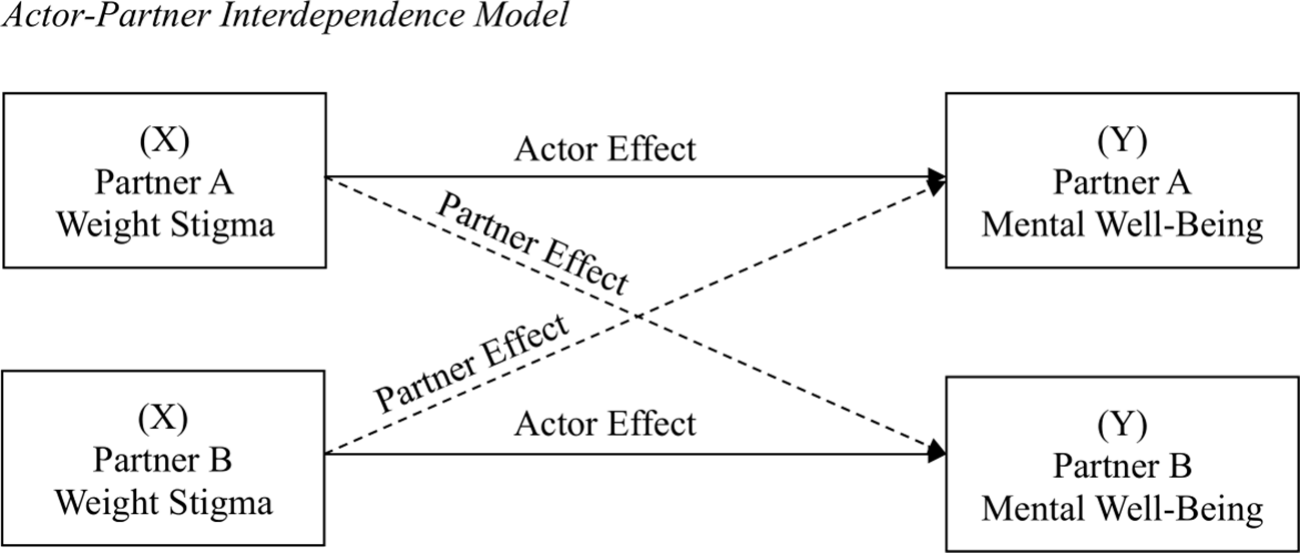
Actor-Partner Interdependence Model.

For the primary analyses, we ran three APIMs to estimate actor and partner effects of internalized, anticipated, and experienced weight stigma on mental well-being. The APIM is the default data analytic method for dyadic data because it integrates appropriate statistical techniques for measuring and testing the interdependence between the two people in the couple (38). The analysis focuses on two variables, the predictor (weight stigma; denoted as X) and the outcome (mental well-being; denoted as Y), that are measured on both members of the romantic pair. In the APIM (see **Figure 2**), paths from a person’s X to the person’s Y are called actor effects, whereas paths from a person’s X to their partner’s Y are called partner effects. To examine sex differences, males were coded as Partner A and females were coded as Partner B in the analyses. Following inclusive practices in relationships research to include all participants in analyses, participants in same-sex relationships were randomly assigned as Partner A or Partner B (50). A sensitivity analysis was conducted with and without participants in same-sex relationships to determine the robustness of effects.

Kenny’s (51) APIM_MM program was used to conduct these analyses. The APIM_MM is based on an R program using R Studio’s Shiny package. The program uses multilevel modeling to estimate the correlation of the errors of the two partners using generalized least squares. The estimates and standard errors produced by the program are identical or very similar to those from conventional multilevel modeling programs. The tests of actor, partner, and covariate effects use a *Z* test. The program creates a sampling distribution of 40,000 cases to obtain confidence intervals. All variables were grand mean centered.

## 3 Results

### 3.1 Preliminary analyses

Descriptive statistics and correlations are provided in **Table 1**. Overall, mean levels of internalized, anticipated, and experienced weight stigma were below the midpoint of the scale and mean levels of mental well-being were above the midpoint of the scale, all *t*s > 8.71, *p*s <.001. Participant scores ranged along the full scales on all measures. Based on mental well-being scores, 220 participants (38%) were classified as flourishing, 307 (54%) as moderate, and 47 (8%) as languishing.

**Table 1:** Descriptive statistics and correlations.

Measures	*M* (*SD*)	1	2	3	4	5	6
1. Internalized Weight Stigma	3.26 (1.61)	.15*					
2. Anticipated Weight Stigma	3.28 (1.99)	.78***	**.11**				
3. Experienced Weight Stigma	0.98 (0.94)	.55***	.59***	.17**			
4. Mental Well-Being	2.91 (1.12)	-.46***	-.34***	-.28***	.43***		
5. BMI	29.44 (7.77)	.52***	.44***	.45***	-.11*	.31***	
6. Self-Perceived Weight	4.76 (1.36)	.65***	.50***	.40***	-.22***	.73***	**.10**

Bolded values along the diagonal represent correlation between partner reports.

*** *p* <.001. ** *p* <.01. * *p* <.05.

Internalized, anticipated, and experienced weight stigma were significantly positively correlated with each other. Internalized, anticipated, and experienced weight stigma were all significantly negatively correlated with mental well-being. BMI and self-perceived weight were significantly positively correlated with internalized, anticipated, and experienced weight stigma, and significantly negatively correlated with mental well-being. A large, positive correlation was observed between BMI and self-perceived weight.

Significant sex and gender differences were observed (see **Table 2**). For sex, females reported more internalized, anticipated, and experienced weight stigma than males. No significant sex differences were observed for mental well-being. For gender, women and gender non-binary participants reported more internalized and anticipated weight stigma than men. Gender non-binary participants reported more experienced weight stigma than women and men, and women reported more experienced weight stigma than men. Gender non-binary participants reported lower mental well-being than men and women.

**Table 2:** Sex and gender differences on mean levels of key variables of interest.

Sex						
Measures	Females (*n* = 301)		Males (*n* = 273)	*t*	*p*	*d*
Internalized Weight Stigma	3.59 (1.62)		2.90 (1.51)	5.25	<.001	0.44
Anticipated Weight Stigma	3.79 (2.00)		2.70 (1.82)	6.82	<.001	0.57
Experienced Weight Stigma	1.13 (0.98)		0.82 (0.88)	3.93	<.001	0.33
Mental Well-Being	2.86 (1.09)		2.97 (1.15)	1.14	.254	0.10
Gender						
	Women (*n* = 289)	Men (*n* = 269)	Non-Binary (*n* = 16)	*F* ratio	*p*	η^2^
Internalized Weight Stigma	3.58_a_ (1.63)	2.90_b_ (1.51)	3.64_a_ (1.66)	13.29	<.001	.08
Anticipated Weight Stigma	3.77_a_ (2.01)	2.71_b_ (1.82)	3.70_a_ (2.06)	21.61	<.001	.11
Experienced Weight Stigma	1.09_a_ (0.97)	0.83_b_ (0.88)	1.59_c_ (0.93)	9.34	<.001	.06
Mental Well-Being	2.88_a_ (1.09)	2.99_a_ (1.15)	2.23_b_ (0.79)	3.66	.026	.03

Standard deviations are presented below means in parentheses. For gender analyses, means with different subscripts significantly differ from each other.

### 3.2 Actor-partner interdependence models

Three analyses were conducted examining the dyadic associations between (1) internalized weight stigma, (2) anticipated weight stigma, and (3) experienced weight stigma and mental well-being. The test of overall distinguishability was not statistically significant in any of the models, indicating that sex did not make a statistically meaningful difference, all X^2^ (4, *N* = 574) < 6.61, *p* >.157. This remained the case when participants in same-sex relationships were excluded from analyses, all X^2^ (4, *N* = 496) < 4.41, *p* >.353. Thus, given that sex did not distinguish the dyadic associations between the variables, dyad members were treated as indistinguishable in the analyses reported below. For APIMs with indistinguishable dyads, models constrain actor paths and partner paths to be equal; therefore, there is one actor path and one partner path to report for each model. BMI was included as a covariate given its significant correlations with weight stigma and mental well-being. Results remained the same when self-perceived weight was included as a covariate instead of BMI. Results are reported on the total scale of the Mental Health Continuum-Short Form. With only one exception (where marginal non-significance was observed), the same pattern of results was observed across the three subscales of emotional, psychological, and social well-being. These analyses are reported in the **Supplementary Material**.

#### 3.2.1 Internalized weight stigma

The APIM examining the dyadic associations between internalized weight stigma and mental well-being showed that internalized weight stigma was negatively associated with participants’ own mental well-being, *B* = -0.37, *SE* = 0.03, *t* = -12.81, *p* <.001, β = -0.54, *r* = -.48 (medium effect size), as well as the mental well-being of their partners, *B* = -0.08, *SE* = 0.02, *t* = -3.23, *p* = .001, β = -0.12, *r* = -.13 (small effect size).

#### 3.2.2 Anticipated weight stigma

The APIM examining the dyadic associations between anticipated weight stigma and mental well-being also showed that anticipated weight stigma was negatively associated with participants’ own mental well-being, *B* = -0.20, *SE* = 0.02, *t* = -8.33, *p* <.001, β = -0.36, *r* = -.33 (medium effect size), as well as the mental well-being of their partners, *B* = -0.05, *SE* = 0.02, *t* = -2.35, *p* = .019, β = -0.09, *r* = .10 (small effect size).

#### 3.2.3 Experienced weight stigma

The APIM examining the dyadic associations between experienced weight stigma and mental well-being showed that experienced weight stigma was negatively associated with participants’ own mental well-being, *B* = -0.33, *SE* = 0.05, *t* = -6.31, *p* <.001, β = -0.28, *r* = -.25 (small effect size), but not significantly associated with the mental well-being of their partners, *B* = -0.05, *SE* = 0.05, *t* = 0.99, *p* = .324, β = -0.04, *r* = -.04.

## 4 Discussion

Although romantic relationships are identified as one of the most frequent and psychologically harmful sources of weight stigma (52, 53), previous research has not yet examined the associations between weight stigma and mental well-being of both romantic partners through a dyadic approach. The current study is the first to examine associations between internalized, anticipated, and experienced weight stigma and mental well-being in couples. The use of APIMs was intended to examine both actor effects, or the impact of weight stigma on one person’s mental well-being, and partner effects, or the impact of that person’s internalization, anticipation, or experience of weight stigma on their partner’s mental well-being.

In alignment with hypotheses and previous research, results demonstrated negative associations between internalized, anticipated, and experienced weight stigma and participants’ own mental well-being. The negative association between weight stigma internalization, or self-derogation based on body weight, and mental well-being is consistent with existing research’s aggregated strong negative association between weight bias internalization and mental health more broadly (54). Pearl and Puhl’s (54) systematic review shows that weight bias internalization is significantly, positively associated with depression, anxiety, disordered eating, and psychological distress, and significantly, negatively associated with self-esteem, body image, and quality of life. The negative association between anticipated weight stigma and participants’ mental well-being can be understood through the social identity threat model as high awareness and expectation of discriminatory treatment based on an identity status typically excluded from power and privilege (55). For example, Hunger et al. (56) found larger-bodied women experience lowered cognitive and cardiovascular performance when anticipating rejection from an anti-fat peer. In general, vigilance toward stigma is linked to internalizing symptoms including depression (57, 58). Vigilance to weight stigma in particular results in behavioral changes like health care avoidance (34) and higher perceived stress as well as oxidative stress (59) that may contribute to mental health difficulties. Lastly, research consistently shows a connection between more frequent experiences of weight stigma and worse mental health, with overall effect sizes estimated as moderate to large (10). Experienced weight stigma negatively affects physical and mental health symptoms through internalized weight stigma and anticipated weight stigma (30, 32, 34, 78).

Results showed that the internalization and anticipation of weight stigma was also negatively associated with the mental well-being of participants’ romantic partners. These results may reflect the relational spillover of weight stigma, as internalization is associated with body shame and self-doubt that may result in withdrawal and loss of intimacy in romantic relationships (8, 9), and thus may undermine partners’ support of each other and further negatively affect mental well-being for both partners. Anticipating weight stigma in general contributes to increased stress and decreased self-esteem (60), which could cross over from one partner in a manner consistent with the concept of dyadic stress in intimate relationships (61). Another possibility, however, is that these results may reflect the relational spillover of partner mental well-being, as partner mental well-being may serve as a protective factor against the internalization or anticipation of weight stigma. Dyadic coping is a powerful protective factor in relationships and well-being (62). Some research suggests that social well-being and connectedness may be protective against the development of internalized weight stigma (63). Stigma by association, or the process through which companions of stigmatized people are socially devalued, offers yet another possible interpretation of the findings (64). The negative association between participants’ internalized and anticipated weight stigma and partners’ mental well-being might reflect, at least in part, partners’ own experiences with stigma by association.

Unexpectedly, these potential relational spillover effects did not extend to experienced weight stigma, as participants’ experienced weight stigma was not significantly associated with their partners’ mental well-being. This finding contrasts with previous research documenting actor and partner effects of experienced stigma on mental health indicators (7, 27). Similar to our findings, in their study of social stigma with gay, lesbian, and bisexual participants, Doyle and Molix (3) found a greater impact of internalized relative to experienced stigma on romantic relationship functioning. Although experiences of weight discrimination and internalized weight stigma are associated with lower psychological well-being in general (10, 12), internalized weight bias has a stronger impact on mental health (i.e., positive affect, negative affect, and self-esteem) than perceived weight discrimination (33). This, in addition to the fact that partner effects sometimes fail to replicate due to relatively small effects (65), might explain why a significant partner effect was not observed for experienced weight stigma in this study.

Significant sex and gender differences were observed in mean levels of the key variables of interest in the current study, such that women generally reported higher levels of internalized, anticipated, and experienced weight stigma than men. Notably, however, no significant sex differences were observed in the dyadic associations between weight stigma and mental well-being. Although women generally report higher internalization of weight bias than men (66) and husbands’ expressions of weight criticism toward their wives has been the focus of research thus far (9), weight stigma is clearly associated with the mental well-being of partners in romantic relationship regardless of sex.

### 4.1 Limitations and future directions

Although the current study recruited a large sample of couples in long-term relationships and demonstrated novel findings regarding the dyadic associations between internalized, anticipated, and experienced weight stigma and mental well-being, some limitations are present that constrain the generalizability of the results. The present study recruited a large sample of couples in long-term relationships and assessed internalized, anticipated, and experienced weight stigma; however, it was cross-sectional which limits the ability to draw conclusions about directionality, temporality, and causality. The sample included people who were diverse in terms of race/ethnicity, gender, sexual identity, age, and body size, although the vast majority of participants were in different-sex relationships and White. Participants’ relationship structures are unknown (e.g., monogamous, non-monogamous, polyamorous). The measures that assessed weight stigma were validated with majority-White samples (30, 31) and thus may not accurately or comprehensively assess weight stigma in diverse racial or ethnic groups. Previous research documents significant racial and gender differences in how weight stigma is internalized and experienced (66). Consequently, the findings from this study may not generalize to people who are not White or not in heterosexual relationships. In addition, the mental well-being of the sample was relatively high, with the majority of participants classified as flourishing or moderately mentally healthy. In addition, weight stigma was not highly internalized, anticipated, or experienced in the sample. It is possible that the results of this study may not generalize to people with lower, languishing levels of mental well-being or higher levels of weight stigma. However, the consistent pattern of actor and partner effects present in a sample that was relatively mentally healthy with lower levels of weight stigma may also highlight the significance of the findings. Finally, although the findings of the present study are important in broadening the field’s understanding of weight stigma and mental well-being among people in romantic relationships, they are novel and yet to be replicated.

These limitations highlight the importance of obtaining longitudinal data in future studies to examine dyadic associations between weight stigma and the mental health of romantic partners, how these constructs evolve over time, and potential relational spillover effects. Such work may seek to examine internalized and anticipated weight stigma as mediators of the association between experienced weight stigma and mental well-being of participants and their romantic partners (30, 32). Additional mechanisms, such as relationship strain (7), affiliate stigma (stigma by association; 11, 64), and relationship and sexual satisfaction (77) are also deserving of future research attention. Future research is encouraged to replicate and expand this work with couples with more diverse demographic characteristics, lower levels of mental well-being, and higher levels of weight stigma to assess the generalizability of this study’s findings. Future research that applies intersectional frameworks to examine people in relationships who are experiencing barriers due to multiple social stigmas (e.g., Black women in lesbian relationships) are especially encouraged given the relatively limited focus of weight stigma research beyond White women (39, 66). Future research may also seek to examine the influence of specific sources of weight stigma (e.g., romantic partner, health care providers, coworkers), as well as potential moderation by weight status and whether couples are matched-weight versus mixed-weight. Finally, future research that examines how romantic partners provide support in coping with weight stigma are encouraged, building off of previous research examining individual strategies to cope with weight stigma (67, 68).

### 4.2 Implications

Despite growing awareness of the negative consequences of weight stigma, previous research on body size and romantic relationships often reinforces harmful stereotypes and assumptions about people in larger bodies. For example, studies have treated romantic relationships as a risk factor for weight gain and romantic partners as an important motivator for weight loss (69), with some researchers endorsing the use of weight stigma to increase health behaviors in couples (e.g., 70). Policy changes are needed to challenge and dismantle weight-normative assumptions. The current climate of healthcare policy rests on the erroneous assumptions that higher body weight results in poorer health, long-term weight loss is widely achievable, and weight loss results in consistent improvement of health outcomes, despite the fact that none of these assumptions are empirically supported (79). The need for policy change is further underscored by the dynamics of weight stigma in close relationships. To this end, institutional and nationwide policies that track relationship variables, partner well-being, and various forms of weight stigma would prove invaluable.

Considering the potential relational spillover of weight stigma and partner well-being in romantic relationships, couples therapists are encouraged to attend to the dyadic influence of internalized and anticipated weight stigma on both partners’ mental health and well-being. This focus emphasizes the urgent need for the development of clinically-oriented strategies to mitigate the relational effects of weight stigma and enhance partner well-being to foster supportive dyadic coping strategies. For example, family and marital clinicians could incorporate weight-bias reduction strategies in their clinical practice, as these efforts show efficacy in a variety of settings (71). Applying clinical principles from acceptance and commitment therapy and cognitive behavioral therapy has also shown effectiveness in reducing weight bias internalization (54, 72). Clinical interventions show efficacy in improving dyadic coping by focusing on the enhancement of coping resources in couples counseling (73). By extending existing individual strategies to address weight stigma and well-being to relational approaches, more inclusive, compassionate, and comprehensive initiatives can be developed. Cook and colleagues (74), for example, highlight the importance of addressing the impact of stigma not only at the individual level but also encouraging meaningful, enriching communication at the interpersonal level, in an effort to challenge biases, foster awareness, and garner support.

Addressing these issues within couples counseling could enhance emotional, psychological, and social dimensions of mental well-being, as conceptualized in Keyes’ (48, 49) and Ryff’s (46) models of psychological well-being. To successfully incorporate themes of bodily autonomy and size inclusivity, therapists are tasked with the challenge of assessing and confronting their own biases, emphasizing the harmfulness of weight stigma in-session, and using non-stigmatizing language in their practice (75). Furthermore, recognition of romantic partners as potential sources of weight stigma, as well as size affirmation, is essential in therapeutic contexts (35, 36, 76). By fostering an environment that prioritizes compassion and inclusion, therapists can help couples build stronger connections, improve communication, and reduce the mental health burdens associated with weight stigma. Incorporating these strategies into clinical practice represents a vital step toward more equitable and effective relationship counseling.

### 4.3 Conclusion

Weight stigma is pervasive, prevalent, and harmful (52). Weight stigma does not occur in a vacuum; it affects people as they live, work, play, and love. Not only is internalized, anticipated, and experienced weight stigma negatively associated with one’s own mental well-being, including emotional, psychological, and social components, but weight stigma, particularly when it is anticipated and internalized, is also negatively associated with the mental well-being of romantic partners. Future research is encouraged to further examine this phenomenon and clinicians are encouraged to adopt weight-inclusive approaches to help people in romantic relationships cope with weight stigma in more psychologically meaningful ways.

## Data Availability

The datasets presented in this study can be found in online repositories. The names of the repository/repositories and accession number(s) can be found below: Open Science Framework https://osf.io/argzt/?view_only=8bcd35aeeb1145c3aa3454cc580db87e.
