# Supplementary material for: Associations between weight stigma and mental well-being among people in romantic relationships: an actor-partner interdependence model investigation
Source: Front Psychiatry. 2025 Jun 6;16:1576406. doi: 10.3389/fpsyt.2025.1576406 (PMC12179220; doi:10.3389/fpsyt.2025.1576406)
Supplement: Supplementary file 1 [file Table1.docx]

***Supplementary Material***

| **Table S1** | | |
| --- | --- | --- |
| *Actor and Partner Effects with Mental Well-Being Subscales* | | |
| Emotional Well-Being | | |
| Internalized Weight Stigma | Actor Effect | *B* = -0.34, *SE* = 0.03, *t* = -11.91, *p* < .001, β = -0.51, *r* = -.45 (medium effect size) |
|  | Partner Effect | *B* = -0.06, *SE* = 0.02, *t* = -2.60, *p* = .010, β = -0.10, *r* = -.11 (small effect size) |
| Anticipated Weight Stigma | Actor Effect | *B* = -0.18, *SE* = 0.02, *t* = -7.73, *p* < .001, β = -0.34, *r* = -.31 (medium effect size) |
|  | Partner Effect | *B* = -0.04, *SE* = 0.02, *t* = -1.72, *p* = .086, β = -0.07, *r* = -.07 |
| Experienced Weight Stigma | Actor Effect | *B* = -0.33, *SE* = 0.05, *t* = -6.57, *p* < .001, β = -0.29, *r* = -.26 (small effect size) |
|  | Partner Effect | *B* = -0.04, *SE* = 0.05, *t* = -0.81, *p* = .420, β = -0.03, *r* = -.03 |
| Psychological Well-Being | | |
| Internalized Weight Stigma | Actor Effect | *B* = -0.40, *SE* = 0.03, *t* = -12.69, *p* < .001, β = -0.54, *r* = -.48 (medium effect size) |
|  | Partner Effect | *B* = -0.08, *SE* = 0.03, *t* = -2.96, *p* = .003, β = -0.11, *r* = -.12 (small effect size) |
| Anticipated Weight Stigma | Actor Effect | *B* = -0.22, *SE* = 0.03, *t* = -8.52, *p* < .001, β = -0.37, *r* = -.34 (medium effect size) |
|  | Partner Effect | *B* = -0.05, *SE* = 0.02, *t* = -2.35, *p* = .019, β = -0.09, *r* = -.10 (small effect size) |
| Experienced Weight Stigma | Actor Effect | *B* = -0.33, *SE* = 0.06, *t* = -5.82, *p* < .001, β = -0.26, *r* = -.24 (small effect size) |
|  | Partner Effect | *B* = -0.01, *SE* = 0.05, *t* = -0.26, *p* = .781, β = -0.01, *r* = -.01 |
| Social Well-Being | | |
| Internalized Weight Stigma | Actor Effect | *B* = -0.36, *SE* = 0.04, *t* = -9.86, *p* < .001, β = -0.43, *r* = -.38 (medium effect size) |
|  | Partner Effect | *B* = -0.09, *SE* = 0.03, *t* = -2.91, *p* = .004, β = -0.11, *r* = -.12 (small effect size) |
| Anticipated Weight Stigma | Actor Effect | *B* = -0.19, *SE* = 0.03, *t* = -6.39, *p* < .001, β = -0.28, *r* = -.26 (small effect size) |
|  | Partner Effect | *B* = -0.06, *SE* = 0.03, *t* = -2.09, *p* = .037, β = -0.08, *r* = -.09 (small effect size) |
| Experienced Weight Stigma | Actor Effect | *B* = -0.33, *SE* = 0.06, *t* = -5.18, *p* < .001, β = -0.23, *r* = -.21 (small effect size) |
|  | Partner Effect | *B* = -0.09, *SE* = 0.06, *t* = -1.59, *p* = .112, β = -0.06, *r* = -.06 |
